# Protective Effects of Cashew Apple Bagasse and Its Hydroethanolic Extract Against Fatty Liver in Rats

**DOI:** 10.3390/antiox15050592

**Published:** 2026-05-07

**Authors:** Susana Hernández-Pérez, Víctor Hugo Oidor-Chan, Jonathan Puente Rivera, Vicente Castrejón-Téllez, Rosa María Oliart-Ros, Elizabeth Carreón-Torres, Luz Ibarra-Lara, Yanet Chávez-Reyes, Diana Catalina Castro-Rodríguez, Patricia Guillermina Mendoza-García, Javier Flores-Estrada, Abril Ramírez-Higuera

**Affiliations:** 1 Food Research and Development Unit, Tecnológico Nacional de México, Campus Veracruz, Veracruz 91897, Mexico; susana61185@gmail.com (S.H.-P.); rosa.or@veracruz.tecnm.mx (R.M.O.-R.); patricia.mg@veracruz.tecnm.mx (P.G.M.-G.); 2 Department of Biotechnology, Universidad Autónoma Metropolitana-Iztapalapa, Mexico City 09340, Mexico; victorhugooidor@xanum.uam.mx; 3 Research Division, Hospital Juárez de México, Mexico City 07760, Mexico; jo_puenter@hotmail.com; 4 Department of Physiology, Instituto Nacional de Cardiología Ignacio Chávez, Mexico City 14080, Mexico; vicente.castrejon@cardiologia.org.mx; 5 Department of Molecular Biology, Instituto Nacional de Cardiología Ignacio Chávez, Mexico City 14080, Mexico; juana.carreon@cardiologia.org.mx; 6 Department of Pharmacology, Instituto Nacional de Cardiología Ignacio Chávez, Mexico City 14080, Mexico; luz.ibarra@cardiologia.org.mx; 7 Department of Food Industry Engineering, Instituto Tecnológico Superior de Las Choapas, Las Choapas 96980, Veracruz, Mexico; yan_chavez@hotmail.com; 8 Department of Immunobiochemistry, National Institute of Perinatology, Mexico City 11000, Mexico; castrodiana182@gmail.com; 9 Institute of Basic Sciences, Universidad Veracruzana, Xalapa-Enríquez 91192, Veracruz, Mexico

**Keywords:** cashew apple bagasse (*Anacardium occidentale* L.), fatty liver disease, antioxidant, inflammation, hepatoprotection

## Abstract

Cashew apple bagasse (*Anacardium occidentale* L.) is an agro-industrial byproduct rich in fiber and phytochemicals, yet its effects on diet-induced fatty liver remain insufficiently characterized. This study evaluated the protective effects of cashew apple bagasse (CAB) and its hydroethanolic extract (HECAB) in rats fed a high-fat, high-carbohydrate (HFHC) diet. The proximate composition of CAB and the phenolic profile and antioxidant capacity of HECAB were characterized. Male Wistar rats were assigned to four groups and fed for 19 weeks with a standard diet, an HFHC diet, or an HFHC diet supplemented with CAB or HECAB. Anthropometric, biochemical, histological, immunohistochemical, and immunoblot analyses were performed. HECAB showed high phenolic content and marked radical-scavenging activity, and untargeted UPLC-QTOF-MS analysis yielded 12 putative secondary metabolite annotations (levels 3–4) based on accurate mass, isotope distributions, MS/MS fragmentation patterns, and predefined acceptance criteria. Relative to the standard diet group, the HFHC diet induced metabolic and hepatic alterations consistent with early-stage MASLD. Compared with HFHC, both CAB and HECAB reduced serum insulin and HOMA-IR, attenuated hepatic steatosis, increased SOD1 and CAT, and reduced NF-κB, IL-6, TNF-α, and IL-1β, whereas GPx1 remained unchanged. Both interventions also enhanced NRF2 and HO-1 compared to HFHC, with stronger nuclear positivity in the HECAB group, while CAB showed the clearest association with IL-10 restoration. These findings are consistent with modulation of antioxidant defense- and inflammatory-related pathways in early-stage MASLD and support further investigation of cashew apple bagasse as a valorized functional ingredient. However, because classical oxidative damage markers were not measured, these results should not be interpreted as direct evidence of reduced oxidative stress. In addition, the detected metabolites should be interpreted as putative annotations rather than definitive compound identifications.

## 1. Introduction

Non-alcoholic fatty liver disease (NAFLD), recently redefined as metabolic dysfunction-associated steatotic liver disease (MASLD), is the most common chronic liver disorder worldwide. It is closely linked to obesity, insulin resistance, and metabolic syndrome [1]. The progression of the disease from simple steatosis to metabolic dysfunction-associated steatohepatitis (MASH) significantly raises the risk of developing cirrhosis, hepatocellular carcinoma, and cardiovascular complications, creating a substantial and growing global health concern [2]. Despite the increasing prevalence of MASLD, treatment options are limited. The primary management strategies include lifestyle modifications such as dietary changes, increased physical activity, and nutritional supplementation. Pharmacological treatments are rare and often have only modest effectiveness or may lead to adverse effects [3].

A large body of evidence supports the idea that oxidative stress and chronic low-grade inflammation play key roles in the development and progression of MASLD. Excessive lipid accumulation in liver cells (hepatocytes) leads to redox imbalance, mitochondrial dysfunction, and activation of inflammatory signaling pathways. These factors together contribute to liver cell damage and disease progression. As a result, dietary antioxidants, especially those derived from plants, have garnered increasing interest for their ability to regulate redox balance and inflammatory processes while offering various beneficial metabolic effects [4,5,6]. Identifying bioactive compounds in foods that can target these intertwined mechanisms represents a promising approach for the prevention and management of MASLD.

The cashew tree (*Anacardium occidentale* L., a member of the Anacardiaceae family) is widely grown in tropical regions and has been recognized for its antimicrobial, antitumor, anti-inflammatory, and hepatoprotective properties [7,8,9,10,11,12,13,14]. The cashew apple (CA), which accounts for nearly 90% of the pseudo-fruit biomass, is rich in dietary fiber, vitamin C, hydrolyzable tannins such as gallic acid, carotenoids, polyphenols, and flavonoids [11,15,16]. However, due to its quick perishability, the cashew apple is often underutilized despite its significant nutritional value [17]. Cashew apple bagasse (CAB), a byproduct of juice extraction that accounts for approximately 20% of the fruit’s weight, is frequently discarded, thereby contributing to agro-industrial waste. Given the large-scale production of cashew apples worldwide, CAB represents an abundant and low-cost source of dietary fiber and bioactive phytochemicals with potential functional value [16,18,19,20].

Previous studies have reported that cashew apple bagasse (CAB) and related cashew-derived products possess antioxidant, antimicrobial, antidiabetic, and anti-inflammatory properties. They may also modulate lipid metabolism, inhibit de novo lipogenesis, and improve insulin sensitivity [21,22,23,24]. However, there is a lack of research examining the biological effects of CAB in models of metabolic liver disease.

This study aimed to analyze the chemical composition of cashew apple bagasse and its hydroethanolic extract. It also evaluated their effects on antioxidant defense pathways, inflammation, and lipid metabolism in a rat model of fatty liver induced by a high-fat, high-carbohydrate diet. The experimental design focused on assessing the biological effects of the hydroethanolic extract as a complex mixture of phytochemicals, rather than isolating individual compounds.

## 2. Materials and Methods

### 2.1. Chemicals and Kits

Folin–Ciocalteu reagent, 2,2-diphenyl-1-picrylhydrazyl (DPPH), 2,2′-Azinobis(3-ethylbenzothiazoline)-6-sulfonic acid (ABTS), Trolox, and analytical standards were obtained from Sigma-Aldrich (St. Louis, MO, USA). Commercial enzymatic kits for measuring glucose, cholesterol, and triglycerides were obtained from Randox Laboratories Ltd. (Crumlin, County Antrim, UK). Insulin was measured using a rat-specific radioimmunoassay (RIA) kit (EMD Millipore, Burlington, MA, USA). Primary antibodies were obtained from Santa Cruz Biotechnology (Dallas, TX, USA), whereas the horseradish peroxidase (HRP)-conjugated secondary antibodies were purchased from Abcam PLC (Cambridge, UK), unless otherwise specified. All solvents used for chromatographic analyses were LC-MS grade. Methanol, acetonitrile, and formic acid were purchased from Merck (Darmstadt, Germany).

### 2.2. Plant Material and Processing of Cashew Apple Bagasse (CAB)

Mature yellow cashew apples (*Anacardium occidentale* L.) were harvested in February 2022 from plantations located in the municipality of Las Choapas, Veracruz, Mexico (17°54′ and 18°12′ N; 95°11′ and 95°32′ W; altitude 10 and 200 m a.s.l.). Botanical identification was performed at the Herbarium of the National Autonomous University of Mexico, and a voucher specimen (No. 8148) was deposited at the Herbarium of the Institute of Ecology A.C. (INECOL).

Cashew apples were separated from the nuts, washed, and processed in accordance with the Mexican sanitary standard NOM-251-SSA1-2009 [25]. Cashew apple bagasse (CAB) was obtained by manually pressing 1 kg of fresh material, freezing at −45 °C, and freeze-drying (36 h, 4.5 Pa; FreeZone^®^ Freeze dryer, Labconco, Kansas City, MO, USA). The dried material was ground using a household grinder (KRUPS coffee mill; Groupe SEB USA, Millville, NJ, USA) and sieved to <0.59 mm (ASTM No. 30). The resulting powder was vacuum-packed and stored at −20 °C until use.

### 2.3. Preparation of the Hydroethanolic Extract of Cashew Apple Bagasse (HECAB)

Thirty-five grams of CAB were mixed with 450 mL of a hydroethanolic solvent (ethanol: water, 50:50 *v*/*v*) at room temperature for three hours. The mixture was then subjected to a pre-clarification step via sedimentation to remove larger particles, followed by filtration through Whatman^®^ No. 1 filter paper (Cytiva, Marlborough, MA, USA). The resulting filtrate was concentrated under reduced pressure using a rotary evaporator (R-1001VN; CHANYO Scientific Instruments Co., Ltd., Zhengzhou, China) at 40 °C. At the end of this process, an aqueous extract was obtained. The extract was stored at −20 °C and protected from light to preserve its stability.

### 2.4. Proximate Composition of Cashew Apple Bagasse (CAB)

The proximate composition of the cashew apple bagasse (CAB) was determined according to the official methods of the Association of Official Analytical Chemists (AOAC, 1996). Moisture content was quantified using the gravimetric method (AOAC method 934.06) and expressed on a dry weight basis. Ash content was determined by controlled incineration to constant weight using method 923.03. Protein content was measured by the Kjeldahl titrimetric method (method 954.01) using a nitrogen-to-protein conversion factor of 6.25. Lipid content was determined via Soxhlet extraction (method 920.39) using petroleum ether as the extraction solvent.fiber was measured using method 993.21, and carbohydrate content was calculated by difference according to Onwuka (2010) [21], using the following formula: % carbohydrate = 100 − (% Fat + % Protein + % Moisture + % Ash).

All analyses were performed in triplicate, and results were expressed on a dry weight basis to ensure accuracy and reproducibility.

### 2.5. Structural Carbohydrates and Lignin Determination

Structural carbohydrates and lignin contents were analyzed according to the procedures described by Fonteles et al. [22], with adaptations for CAB samples. CAB samples were dried to about 10% moisture, and total solids were measured. Acid hydrolysis was performed with 72% (*w*/*w*) sulfuric acid for 1 h, with manual stirring, then diluted to 4% with deionized water and autoclaved at 121 °C for 1 h. The hydrolyzed mixture was vacuum filtered through pre-weighed crucibles for lignin quantification, and the hydrolysate was analyzed at 240 nm using a UV-Vis spectrophotometer (UV-1800 Standalone UV-Vis Double-Beam Spectrophotometer, Shimadzu Corporation, Kyoto, Japan). The solids were dried at 105 ± 3 °C, combusted to determine ash content, and the cellulose and hemicellulose were quantified from the hydrolysate by neutralizing to pH 5–6 with calcium carbonate. The filtered supernatant was analyzed using an HPLC system (Waters Corp., Milford, MA, USA) equipped with a refractive index detector (Waters Corp., Milford, MA, USA) and a Shodex SH1011 column (Showa Denko K.K., Tokyo, Japan) at 55 °C, with 0.05 N H_2_SO_4_ as the mobile phase at 0.6 mL/min. Quantification was performed using calibration curves and Empower software (version 2.0; Waters Corp., Milford, MA, USA) for data processing.

### 2.6. Determination of Total Polyphenols Content in HECAB

Total phenolic content was determined using the Folin–Ciocalteu method as described by Prior et al. [26]. Antioxidant capacity was evaluated using the DPPH and ABTS•+ radical-scavenging assays according to established protocols [19,27]. Results were expressed as mg gallic acid equivalents (GAE) per 100 g of dry extract for TPC and as µmol Trolox equivalents (TE) per g of dry extract for antioxidant capacity. For the ABTS•+ assay, antioxidant capacity values were calculated using the appropriate sample dilution factor applied at the time of measurement. All assays were performed in triplicate.

### 2.7. Identification of Polyphenols via Ultra-Performance Liquid Chromatography Coupled to Mass Spectrometry (UPLC-QTOF-MS)

Polyphenolic compounds in HECAB were analyzed by UPLC-QTOF-MS using a previously described untargeted UHPLC-HRMS metabolomics workflow, with minor modifications tailored to the present extract and instrument settings [28].

For chromatographic analysis, lyophilized HECAB was dissolved in distilled water at 10 mg/mL and stored in the dark at 4 °C for 3 days to ensure complete solubilization. Prior to analysis, samples were filtered through a 0.22 μm PTFE membrane. Polyphenol separation was performed using an ACQUITY UPLC H-Class Plus Bio System (Waters Corp., Milford, MA, USA) equipped with a binary solvent manager, autosampler, and column oven. Chromatographic separation was achieved on an ACQUITY UPLC BEH C18 column (100 mm × 2.1 mm, 1.7 μm particle size). The mobile phases consisted of phase A, LC-MS-grade water containing 0.01% formic acid, and phase B, LC-MS-grade acetonitrile containing 0.01% formic acid. The flow rate was set at 0.3 mL/min, and the injection volume was 10 μL. The gradient elution program was as follows: 0–9 min, 96% phase A; 10–14 min, 80% phase A; 15–19 min, 65% phase A; 20–24 min, 100% phase B; 25–29 min, 100% phase B; followed by a 30 min re-equilibration period at 96% phase A.

Mass spectrometric detection was performed using a Xevo G2-XS QTOF mass spectrometer (Waters Corp., Milford, MA, USA) equipped with an electrospray ionization (ESI) source operating in negative ion mode. Data acquisition and instrument control were carried out using UNIFI software (version 1.9.4; Waters Corp., Milford, MA, USA). The MS operating parameters were as follows: capillary voltage, 2.0 kV; cone gas (N_2_), 400 L/h; desolvation gas (N_2_), 50 L/h; source temperature, 150 °C; and desolvation temperature, 500 °C. Lock-mass correction was performed using leucine enkephalin (200 pg/mL in acetonitrile–water, 50:50, *v*/*v*, containing 0.1% formic acid). Instrument calibration was conducted using 0.5 mM sodium formate prepared in 2-propanol–water (90:10, *v*/*v*), covering a mass range of *m*/*z* 50–1200 with a scan time of 0.250 s. Data were acquired in MSE mode, using a low collision energy of 6 eV and a high-energy ramp from 20 to 30 eV. The cone voltage was set at 20 V. All solvents and reagents used were LC-MS grade.

Raw LC-MS data were subsequently processed using Progenesis QI software (version 2.3; Nonlinear Dynamics, Newcastle, UK). Data processing included baseline correction, peak detection and integration, retention time alignment, peak matching, and normalization. Putative metabolite annotation was based on accurate mass-to-charge ratio (*m*/*z*), isotope distribution, and MS/MS fragmentation patterns. The reference databases used for annotation included LuMet-TCM, Animal_DB, and Herb DB. All compound assignments were considered putative annotations and were accepted when the overall identification score was ≥40, and the MS/MS fragmentation match score exceeded 50. For semi-quantitative analysis, the relative peak areas of individual metabolites were normalized to 100% to generate the final data matrix. Individual metabolites were not isolated or tested separately; therefore, their contribution to the observed biological effects is interpreted as part of the overall extract composition.

### 2.8. Animal Model and Experimental Design

Experimental procedures were approved by the Ethics Committee for Animal Studies of the Technological Institute of Veracruz (protocol code CI-ITVER/12/2021; approval date: 12 August 2021) and were conducted according to NOM-062-ZOO-1999 and the International Ethical Guidelines for Biomedical Research Involving Laboratory Animals, and the ARRIVE 2.0 guidelines [29].

To reduce variability associated with estrous cycle-related hormonal fluctuations that can influence metabolic and inflammatory readouts, 32 male Wistar rats (10 weeks, 280 ± 10 g) were used, which were housed under controlled environmental conditions (12 h light/12 h dark cycle, 24 ± 2 °C, 50–60% humidity). The animals were randomly divided into four groups (*n* = 8 per group): CTRL: standard diet (Lab Diet 5001, LabDiet^®^, St. Louis, MO, USA); HFHC: high-fat, high-carbohydrate diet-fed rats; CAB: HFHC supplemented with 10% *w*/*w* cashew apple bagasse pellets; HECAB: HFHC + hydroethanolic extract of CAB administered orally at 1 mL/100 g body weight. Food and water were supplied *ad libitum* during the 19-week experimental period. The HFHC diet was prepared as described by Panchal et al. [30] with minor modifications.

### 2.9. Biochemical Analysis

During week 19, after a 12 h overnight fast, animals were euthanized by rapid decapitation, and blood samples were immediately collected. Decapitation was selected because it allows rapid loss of consciousness and immediate sample collection without the potential biochemical alterations induced by anesthetic agents. This method is recognized as an acceptable euthanasia technique for small rodents when performed by trained personnel according to the NOM-062-ZOO-1999 and the guidelines of the American Veterinary Medical Association.

Serum was collected via centrifugation (1449× *g* for 15 min at room temperature), and the medial lobe of the liver was excised and divided into several sections. These sections were then frozen and stored at −70 °C, while others were fixed in neutral formalin.

The serum concentrations of glucose, total cholesterol, and triglycerides were determined using commercial enzymatic colorimetric assays according to the manufacturer’s instructions (Glucose PAP, Cat. No. GL3815; Cholesterol, Cat. No. CH3810; Triglycerides, Cat. No. TR3823; RANDOX Laboratories Ltd., Crumlin, County Antrim, UK). Serum insulin levels were measured using a rat-specific radioimmunoassay (RIA) kit provided by Linco Research, Inc./EMD Millipore, St. Charles, MO, USA. Insulin resistance was estimated using the HOMA-IR index, as previously described [31].

### 2.10. Histological and Immunohistochemical Assays

To evaluate the MASLD score, tissue sections were stained with hematoxylin and eosin (H&E) and assessed using the semiquantitative scoring system developed by the NASH Clinical Research Network (NASH CRN) Pathology Committee. This scoring system evaluates three components: steatosis (0–3), lobular inflammation (0–3), and hepatocellular ballooning (0–2). The MASLD activity score (NAS) is calculated as the unweighted sum of these components, yielding a total score ranging from 0 to 8 [32].

Adjacent sections were stained according to the picrosirius red stain kit (ab150681; Abcam PLC, Cambridge, UK). Fibrosis was quantified as the collagen deposition proportional area (CPA), calculated as the percentage of picrosirius red-positive collagen area relative to the total tissue area, using the formula: CPA (%) = (collagen-positive area/total tissue area) × 100.

For immunohistochemical assays, liver sections were prepared according to the procedures described by Flores-Estrada J et al. [33]. The following primary antibodies were used: nuclear factor erythroid 2-related factor (NRF2; sc-722; Santa Cruz Biotechnology Inc., Dallas, TX, USA), heme oxygenase 1 (HO-1; sc-7695), and nuclear factor κ-light-chain-enhancer of activated B cells p65 subunit (NF-κBp65; sc-8008). Primary antibodies were applied at dilutions ranging from 1:50 to 1:200 and incubated overnight at 4 °C. Sections were then incubated with horseradish peroxidase (HRP)-conjugated secondary antibodies (1:100; goat anti-rabbit or goat anti-mouse) for 2 h at room temperature. Detection was performed using DAB chromogen (3,3′-diaminobenzidine, K047; Diagnostic BioSystems, Pleasanton, CA, USA), followed by hematoxylin counterstaining.

For histological and immunohistochemical assessments, images were captured using bright-field microscopy (Carl Zeiss Axio imager.A2 microscope; Carl Zeiss Microscopy GmbH, Jena, Germany) equipped with an Axiocam ICc5 camera and ZEN Microscopy Software (version 2.3; Carl Zeiss Microscopy GmbH, Oberkochen, Germany) with fixed threshold settings. Two independent observers, blinded to the animal’s identity, analyzed 10 non-overlapping microscopic fields per animal at 200× magnification. The percentage of immune-positive cells was calculated as the ratio of stained cells (nuclei or cytoplasmic positivity, as appropriate) to the total number of cells per field.

### 2.11. Western Blot Assays

Frozen liver samples were ground in liquid nitrogen. The resulting tissue powder was resuspended in lysis buffer (25 mM HEPES, 100 mM NaCl, 10% glycerol, 1% Triton X-100, 7 mg/mL sodium deoxycholate, pH 7.5) with a protease inhibitor cocktail (1 mM PMSF, 10 µg/mL pepstatin A, leupeptin, and aprotinin). After incubating at 4 °C for 1 h, the mixture was centrifuged, and the supernatant was collected. Protein concentration was measured using the Bradford assay, and 50 µg of total protein was separated via SDS-PAGE on 8–15% polyacrylamide gels and transferred to PVDF membranes (0.22 μm; Millipore, Burlington, MA, USA). Membranes were blocked with TBS-T and 5% non-fat dry milk. The membranes were then incubated overnight at 4 °C with primary antibodies (1:1000 dilution) against the following proteins: peroxisome proliferator-activated receptor gamma coactivator 1-alpha (PGC-1α; sc-517380), nuclear factor erythroid 2-related factor 2 (NRF2; sc-722), superoxide dismutase 1 (SOD1; sc-271014), catalase (sc-50508), glutathione peroxidase 1 (GPX1; sc-133160), NF-κB p65, tumor necrosis factor-alpha (TNF-α; sc-33639), interleukin-1 beta (IL-1β; sc-7884), interleukin-6 (IL-6; sc-57315), and interleukin-10 (IL-10; sc-32815), followed by incubation overnight at 4 °C with HRP-conjugated secondary antibodies (1:10,000 dilution). Glyceraldehyde-3-phosphate dehydrogenase (GAPDH; sc-365062, Santa Cruz Biotechnology) was used as the loading control. Immunoreactive bands were visualized using the ClarityTM Western ECL substrate kit (Bio-Rad Laboratories, Inc., Hercules, CA, USA) and exposed to X-ray films (AGFA HealthCare, Ortho CP-GU, Mortsel, Belgium).

Densitometric analysis was performed using a GS-800 densitometer (Bio-Rad Laboratories, Hercules, CA, USA), and band intensities were quantified with Quantity One software (version 4.6.9; Bio-Rad Laboratories Inc., Hercules, CA, USA). Protein expression levels were expressed as arbitrary units normalized to GAPDH.

### 2.12. Statistical Analysis

The individual animal was considered the experimental unit (*n* = 8 per group). Anthropometric, biochemical, immunohistochemical, immunoblot, and collagen proportional area (CPA) data are presented as mean ± standard deviation (SD) and were analyzed using one-way ANOVA followed by Tukey’s post hoc test. Ordinal histological endpoints derived from hematoxylin and eosin staining, including the MASLD activity score, are presented as median and interquartile range (IQR) and were analyzed using the Kruskal–Wallis test followed by Dunn’s multiple comparisons post hoc test with Holm adjustment. Statistical significance was set at *p* < 0.05. All analyses were performed using GraphPad Prism software (version 8.0; GraphPad Software, San Diego, CA, USA).

## 3. Results

### 3.1. Proximate and Structural Carbohydrate Composition

Cashew apple bagasse (CAB) exhibited a total carbohydrate content of 49.7 ± 0.8%, of which 11 ± 0.6% corresponded to dietary fiber. The structural fiber fraction consisted predominantly of hemicellulose (35.85 ± 1.50%), followed by cellulose (16.57 ± 0.90%), and lignin (9.37 ± 0.34%). Carbohydrate content was calculated by difference after determination of total protein (30.0 ± 0.9%), moisture (3.0 ± 0.18%), fat (5.5 ± 0.5%), and ash (0.83 ± 0.05%) content.

### 3.2. Total Phenolic Content and Radical-Scavenging Capacity of HECAB

The total phenolic content of HECAB was 6472 ± 773.47 mg gallic acid equivalents (GAE)/100 g of dry extract. In the ABTS•+ assay, HECAB showed a radical-scavenging activity of 1499.43 ± 61.99 µmol Trolox equivalents (TE)/g of dry extract. In the DPPH• assay, HECAB exhibited a radical-scavenging capacity of 1509.66 ± 13.28 µmol TE/g of dry extract. In addition, the percentage of radical inhibition increased in a concentration-dependent manner, reaching 93% in the DPPH• assay and 85% in the ABTS•+ assay.

### 3.3. Phytochemical Profile of the Hydroethanolic Extract of Cashew Apple Bagasse (HECAB)

UPLC-MS analysis yielded putative annotations for 12 secondary metabolites in HECAB based on accurate mass measurements and characteristic MS/MS fragmentation patterns (Table 1). The detected compounds belonged to several structural classes, including flavonoids and isoflavonoids, coumarins and dicoumarins, quinones, chalcones, phenolic acids and esters, tannins, and terpenoids. Representative putatively annotated LC-MS features included signals tentatively assigned to ellagic acid (a hydrolyzable tannin), dicumarol, and artemetin. Because this was an untargeted metabolomic analysis, these assignments should be interpreted as putative rather than definitive identifications, and selected LC-MS features without targeted analytical confirmation were conservatively retained as untargeted annotations. No absolute quantification of individual compounds was performed.

### 3.4. Effects of CAB and HECAB on Anthropometric and Biochemical Parameters in HFHC-Fed Rats

Anthropometric and biochemical parameters are summarized in Table 2. Rats in the CTRL group showed higher food consumption and final body weight than HFHC-fed rats, whereas Lee index values did not differ significantly among groups. Supplementation with CAB or HECAB reduced final body weight by 18% and 8%, respectively, compared with the HFHC group. In addition, energy intake was higher in the HFHC group than in CTRL and was lower in both supplemented groups than in HFHC. The HFHC diet significantly increased serum total cholesterol, glucose, insulin levels, and the HOMA-IR index compared with the CTRL group. CAB and HECAB supplementation markedly reduced serum insulin levels and HOMA-IR values relative to HFHC, reaching values comparable to those of the CTRL group. In contrast, serum total cholesterol remained elevated in both supplemented groups, and glucose levels did not decrease compared with the HFHC group.

### 3.5. Histological Analyses of Liver Tissue from HFHC-Fed Rats Supplemented with Cashew Apple Bagasse (CAB) or Its Hydroethanolic Extract (HECAB)

**Figure 1:**
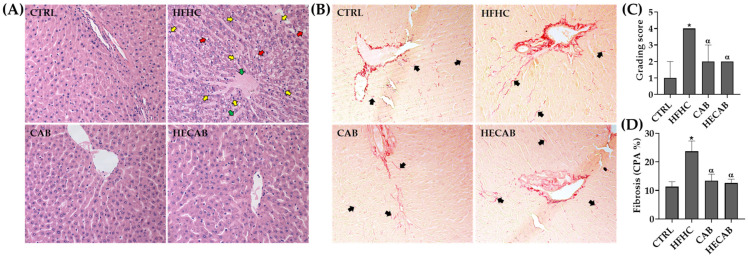
Effect of cashew apple bagasse (CAB) and hydroethanolic extract (HECAB) supplementation on liver histology in HFHC-fed rats. (**A**) Representative hematoxylin and eosin-stained liver sections showing steatosis, ballooning hepatocytes, inflammatory cell infiltration, and vascular congestion. (**B**) Representative Picrosirius Red-stained liver sections showing collagen deposition. (**C**) MASLD activity score in the experimental groups (CTRL, HFHC, CAB, and HECAB), expressed as median and interquartile range (IQR). Statistical analysis was conducted using the Kruskal–Wallis test, followed by Dunn’s multiple comparisons post hoc test with Holm’s adjustment. (**D**) Collagen proportional area (CPA, %), expressed as mean ± SD. Statistical analysis was performed using one-way ANOVA followed by Tukey’s post hoc test (*p* < 0.05). Red, yellow, and green arrows indicate ballooning hepatocytes, inflammatory cells, and vascular congestion, respectively; black arrows indicate collagen deposits. * *p* < 0.05 vs. CTRL; α *p* < 0.05 vs. HFHC. Magnification: 200×.

Representative liver histology images are presented in Figure 1A. The control (CTRL) animals exhibited a normal hepatic architecture with no signs of lipid accumulation or inflammation. In contrast, rats fed a high-fat, high-carbohydrate (HFHC) diet showed significant steatosis, hepatocellular degeneration, and infiltration of inflammatory cells.

Supplementation with CAB or HECAB significantly preserved the hepatic architecture and reduced lipid accumulation and inflammatory infiltration. Semi-quantitative analysis indicated that the HFHC group had markedly higher steatosis and lobular inflammation scores compared to the CTRL animals (*p* < 0.01). Both supplemented groups displayed significantly reduced scores (*p* < 0.01) (Figure 1C). Sirius Red staining revealed increased collagen deposition in HFHC-fed rats (Figure 1B). In contrast, no significant differences in collagen proportional area were found among the CTRL, CAB, and HECAB groups (Figure 1D).

### 3.6. Effects of CAB and HECAB on Endogenous Antioxidant Protein Expression in Fatty Liver

**Figure 2:**
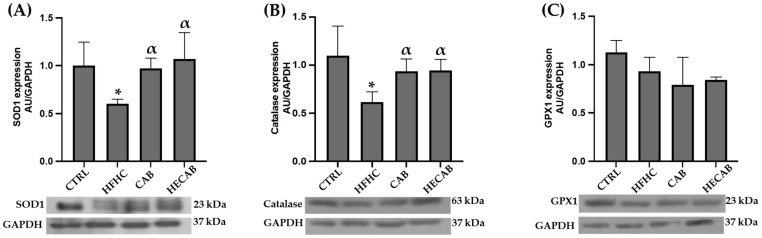
Effects of CAB and HECAB on endogenous antioxidant protein expression in the liver of rats fed a high-fat, high-carbohydrate (HFHC) diet. (**A**–**C**) Densitometric quantification and representative immunoblots of superoxide dismutase 1 (SOD1), catalase, and glutathione peroxidase 1 (GPX1), respectively, in the CTRL, HFHC, CAB, and HECAB groups. Data are expressed as mean ± SD. Statistical analysis was performed using one-way ANOVA followed by Tukey’s post hoc test (*p* < 0.05). * *p* < 0.05 vs. CTRL; α *p* < 0.05 vs. HFHC.

Immunoblot analysis showed that rats fed a HFHC diet had significantly lower levels of hepatic superoxide dismutase 1 (SOD1) and catalase than the CTRL group (Figure 2A,B). Supplementation with CAB and HECAB restored antioxidant protein expression, resulting in levels higher than those in the HFHC group. In contrast, glutathione peroxidase 1 (GPX1) protein levels did not differ significantly among the experimental groups (Figure 2C).

### 3.7. Effects of CAB and HECAB on Cytoprotective Factors in Fatty Liver

**Figure 3:**
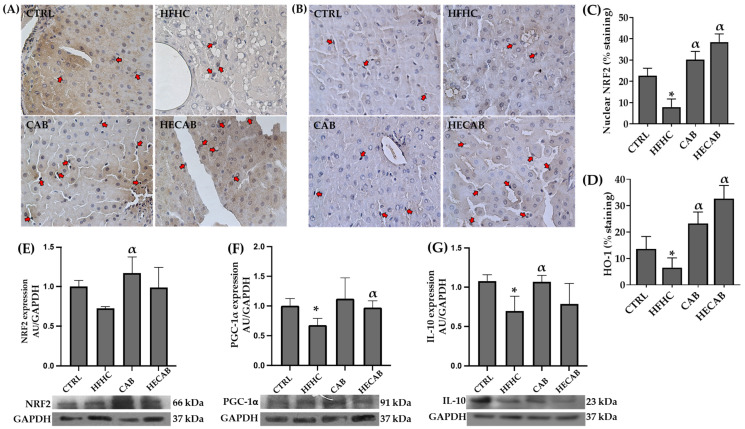
Effects of CAB and HECAB on cytoprotective factor expression in fatty liver. (**A**,**B**) Representative liver sections showing nuclear immunostaining for nuclear factor erythroid 2-related factor 2 (NRF2) and heme oxygenase-1 (HO-1), respectively. (**C**,**D**) Quantitative analysis of NRF2- and HO-1-positive staining, respectively. (**E**–**G**) Protein expression levels of NRF2, peroxisome proliferator-activated receptor gamma coactivator 1 alpha (PGC-1α), and IL-10 in the CTRL, HFHC, CAB, and HECAB groups. Red arrows indicate positive nuclear staining in non-parenchymal cells. Data are expressed as mean ± SD (*n* = 8). Statistical analysis was performed using one-way ANOVA followed by Tukey’s post hoc test (*p* < 0.05). * *p* < 0.05 vs. CTRL; α *p* < 0.05 vs. HFHC. Magnification: 200×.

To determine whether CAB and HECAB modulate the cytoprotective pathway in fatty liver, the expression of nuclear factor erythroid 2-related factor 2 (NRF2) and heme oxygenase-1 (HO-1), as well as the percentage of cells exhibiting nuclear immunoreactivity for these proteins, was evaluated in rats fed an HFHC diet (Figure 3A–E).

HFHC-fed rats exhibited a marked reduction in nuclear localization of NRF2 and HO-1 compared with CTRL animals (Figure 3A–D). Supplementation with CAB significantly increased nuclear positivity for both proteins, whereas HECAB supplementation produced an even more pronounced effect, particularly in hepatocytes and non-parenchymal sinusoidal cells.

Consistent with these findings, immunoblot analysis showed reduced NRF2 protein expression in HFHC-fed rats compared with the CTRL group, whereas CAB and HECAB supplementation increased NRF2 protein expression (Figure 3E). We also assessed the expression of peroxisome proliferator-activated receptor gamma coactivator 1 alpha (PGC-1α) and interleukin-10 (IL-10). Both proteins were significantly reduced in the HFHC group compared with the CTRL group. In the supplemented groups, PGC-1α expression was better preserved in the HECAB group compared to HFHC, whereas the effect in CAB was less marked. In contrast, IL-10 restoration was more evident in the CAB group than in the HECAB group (Figure 3F,G).

### 3.8. Effects of CAB and HECAB on Proinflammatory Cytokine Expression

**Figure 4:**
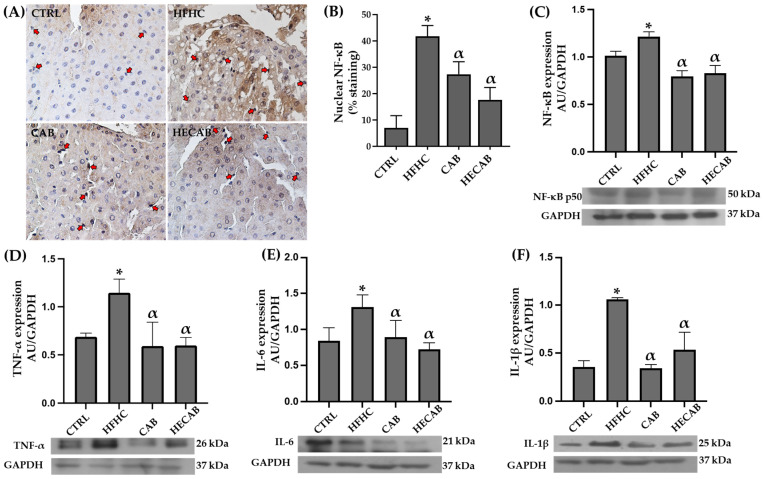
Effects of cashew apple bagasse (CAB) and its hydroethanolic extract (HECAB) on proinflammatory cytokine expression in fatty liver. (**A**) Representative liver sections showing nuclear immunostaining for NF-κB p65. (**B**) Quantitative analysis of NF-κB p65-positive nuclear staining. (**C**–**F**) Protein expression levels of NF-κB p65, TNF-α, IL-6, and IL-1β, respectively, in the CTRL, HFHC, CAB, and HECAB groups. Red arrows indicate positive nuclear staining in non-parenchymal cells. Data are expressed as mean ± SD. Statistical analysis was performed using one-way ANOVA followed by Tukey’s post hoc test (*p* < 0.05). * *p* < 0.05 vs. CTRL; α *p* < 0.05 vs. HFHC.

To evaluate the effects of CAB and HECAB on inflammatory signaling in rats with HFHC diet-induced fatty liver, we assessed NF-κB p65 protein expression and nuclear localization in liver tissue. The HFHC group showed significantly higher NF-κB p65 levels compared to the CTRL, CAB, and HECAB groups. Both CAB and HECAB groups exhibited lower NF-κB expression than CTRL, with no significant difference between them (Figure 4C).

Immunohistochemical analysis indicated that CTRL livers had a lower percentage of NF-κB-positive nuclei than the supplemented groups. In contrast, the HFHC group had the highest percentage, predominantly in non-parenchymal cells. In contrast, nuclear localization in CTRL and supplemented groups was mainly in hepatocytes. Immunoblot analysis confirmed reduced NF-κB in the presence of CAB or HECAB (Figure 4A,B).

Moreover, elevated NF-κB expression and nuclear translocation correlate with increased proinflammatory cytokines. Protein levels of TNF-α, IL-6, and IL-1β were notably higher in the HFHC group compared to CTRL, CAB, and HECAB groups (Figure 4D–F, respectively).

## 4. Discussion

A growing body of evidence indicates that oxidative stress and chronic inflammation are not merely consequences of hepatic steatosis but active drivers of MASLD progression. Excessive lipid accumulation enhances mitochondrial β-oxidation overload, peroxisomal activity, and cytochrome P450-mediated oxidation, thereby increasing the production of reactive oxygen species (ROS). When ROS generation exceeds the detoxification capacity of endogenous antioxidant systems—such as superoxide dismutase (SOD), catalase (CAT), and glutathione peroxidase (GPx)—a state of persistent redox imbalance develops, promoting lipid peroxidation, protein oxidation, DNA damage, and activation of pro-inflammatory signaling pathways [34]. In this context, the nuclear factor erythroid 2-related factor 2 (NRF2) pathway represents a master regulator of cellular antioxidant responses and an important determinant of MASLD susceptibility and progression [35].

Under basal conditions, NRF2 is retained in the cytoplasm by Kelch-like ECH-associated protein 1 (Keap1), which targets it for proteasomal degradation. Oxidative stress induces conformational modifications in Keap1 cysteine residues, thereby stabilizing NRF2 and promoting nuclear translocation [35]. Once in the nucleus, NRF2 binds to antioxidant response elements (AREs), promoting transcription of phase II detoxifying and antioxidant genes, including heme oxygenase-1 (HO-1), NAD(P)H quinone oxidoreductase 1 (NQO1), SOD, CAT, and GPx. Impaired NRF2 signaling has been associated with increased oxidative damage and exacerbated steatohepatitis, whereas pharmacological or nutritional NRF2 activation confers hepatoprotection in experimental MASLD [36,37,38].

We acknowledge that direct measurements of classical oxidative damage markers, such as malondialdehyde and 8-hydroxy-2′-deoxyguanosine, were not included in this study. Therefore, our interpretation of antioxidant modulation is based on changes in endogenous markers of the antioxidant response rather than on direct quantification of oxidative damage [38,39]. Within that scope, CAB and HECAB supplementation increased SOD1 and CAT expression, whereas GPx did not differ significantly among groups. In parallel, both treatments enhanced NRF2 and HO-1 expression. Taken together, these findings are consistent with partial reactivation of endogenous cytoprotective pathways altered during diet-induced steatosis, rather than direct evidence of reduced oxidative damage [40].

Importantly, the NRF2–HO-1 axis extends beyond redox regulation and participates in metabolic control. HO-1 enzymatic activity generates reactive metabolites, such as carbon monoxide (CO), biliverdin, and bilirubin, which exert anti-inflammatory and cytoprotective effects. CO has been shown to activate p38 MAPK and STAT3 signaling cascades, promoting transcription of interleukin-10 (IL-10), a potent anti-inflammatory cytokine with hepatoprotective properties [41,42,43]. IL-10 suppresses pro-inflammatory cytokine production, inhibits macrophage activation, and limits hepatocellular injury in experimental steatohepatitis.

Consistent with this framework, CAB supplementation increased hepatic IL-10 expression in parallel with NRF2/HO-1 activation. These data are consistent with a shift toward an anti-inflammatory hepatic phenotype, although they do not, by themselves, establish a direct causal NRF2–HO-1–IL-10 axis in this model [44,45,46]. Because NRF2-mediated IL-10 regulation has been characterized mainly in immune cells, the contribution of hepatic non-parenchymal cells in our model remains a plausible but unresolved component of this response.

Conversely, the NF-κB pathway—one of the central mediators of inflammation in MASLD—was strongly activated in HFHC-fed rats. Oxidative stress facilitates NF-κB translocation via redox-sensitive kinases, promoting nuclear accumulation of the p65 subunit and transcription of pro-inflammatory cytokines such as TNF-α, IL-6, and IL-1β [47,48,49]. These cytokines exacerbate insulin resistance, stimulate hepatic lipogenesis, and sustain inflammatory cell recruitment. CAB and HECAB reduced NF-κB nuclear localization and lowered cytokine expression, a pattern consistent with coordinated modulation of NRF2- and NF-κB-related signaling rather than proof of a direct reciprocal mechanism [50,51].

Additionally, PGC-1α expression was reduced in HFHC-fed rats, whereas HECAB showed a more evident preservation of its expression relative to HFHC, with a less marked effect in CAB. Therefore, the response of PGC-1α should not be described uniformly across both supplemented groups. Although these data do not support the activation of a PGC-1α-driven pathway, they are consistent with mitochondrial adaptive responses, particularly in the HECAB group. In this context, PGC-1α has been linked to NRF2 regulation through AMPK-dependent mechanisms, while NRF2 may also contribute to mitochondrial adaptive responses. Therefore, the concurrent improvement in antioxidant response-related markers, insulin sensitivity, and steatosis observed in our model is consistent with partial engagement of a PGC-1α/NRF2/HO-1-associated cytoprotective network, rather than definitive activation of this axis [44,45,46].

At the compositional level, CAB obtained from Mexican cashew orchards exhibited a high content of structural carbohydrates (hemicellulose, cellulose, and lignin) and dietary fiber, together with moderate protein and fat fractions. These characteristics are consistent with previous reports that identify cashew apple by-products as fiber-rich matrices with prebiotic, metabolic, and anti-inflammatory potential [18,19,21,22,23].

Our UPLC–QTOF-MS analysis yielded putative annotations for 12 secondary metabolites in HECAB (Table 1). Several of these compounds belong to chemical classes commonly associated with antioxidant and anti-inflammatory activities, supporting the interpretation of HECAB as a polyphenol-enriched extract with multitarget biological potential [16,17,18,21,22,23,24,52,53,54]. However, because these assignments were obtained by means of untargeted UPLC-QTOF-MS and were not confirmed using targeted analytical methods, the detected metabolites should be interpreted as putative annotations rather than definitive compound identifications. Therefore, the biological effects observed in the present model are more appropriately discussed in relation to the overall phytochemical profile of HECAB rather than any single metabolite. Future targeted studies will be necessary to validate and quantify these metabolites and to better define their contribution to the overall phytochemical profile of HECAB.

Overall, our findings suggest that CAB and HECAB modulate antioxidant defense-related and inflammation-related pathways in this model of diet-induced fatty liver. The data support changes in endogenous antioxidant response markers, attenuation of inflammatory signaling, and improvement in insulin resistance; however, because classical oxidative damage markers were not assessed, these findings should not be interpreted as direct evidence of reduced oxidative stress. Accordingly, the present results are more appropriately interpreted as evidence of modulation of antioxidant defense-associated responses, rather than as proof of direct attenuation of oxidative injury or oxidative damage. Furthermore, the present experimental design does not allow us to distinguish whether the observed changes in antioxidant response-related markers reflect true mitigation of oxidative injury by the treatments or an endogenous adaptive response to the HFHC condition. It is important to emphasize that the biological effects observed in this study are attributed to the hydroethanolic extract as a whole. While phytochemical profiling provides valuable insight into its composition, the contribution of individual metabolites cannot be established under the current experimental design and is likely to reflect synergistic or additive interactions within the extract matrix.

## 5. Conclusions

CAB and HECAB exerted hepatoprotective effects in this diet-induced rat model of fatty liver, as evidenced by reduced hepatic steatosis, attenuation of inflammatory signaling, and modulation of antioxidant response-related markers. These findings support the biological relevance of cashew apple bagasse as a valorized agro-industrial byproduct with potential functional application. However, the scope of the present study should be interpreted with caution. Because classical oxidative damage markers were not assessed, the results should not be considered direct evidence of reduced oxidative stress or direct attenuation of oxidative injury, but rather of modulation of antioxidant defense-related pathways and associated inflammatory responses. Likewise, the phytochemical profile of HECAB should be interpreted conservatively, since the detected metabolites correspond to putative level 3–4 annotations obtained by untargeted UPLC-QTOF-MS and were not confirmed using targeted analytical methods, authentic standards, or individual compound isolation. Therefore, the observed biological effects cannot be attributed to specific metabolites and are more appropriately understood in the context of the overall extract matrix.

## Figures and Tables

**Table 1:** Phytochemical profile of metabolites putatively annotated in the hydroethanolic extract of cashew apple bagasse (HECAB) by means of UPLC-MS.

No.	Peak Area Ratio (%)	Retention Time (min)	*m*/*z*	Mass Error (ppm)	Chemical Structural Formula	Metabolites; (IUPAC)
1	84.06	18.68	295.1278	34.5	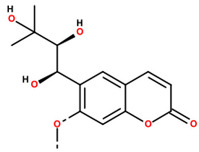	Angelitriol; (7-methoxy-6-[(1R,2S)-1,2,3-trihydroxy-3-methylbutyl]chromen-2-one)
2	77.18	0.72	273.0496	−95.6	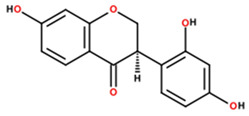	2,4’,7-trihydroxyisoflavanone; ((3*R*)-3-(2,4-dihydroxyphenyl)-7-hydroxy-2,3-dihydrochromen-4-one)
3	68.01	17.68	289.1099	10	Not assigned	Unassigned LC-MS feature (*m*/*z* 289.1099; RT 17.68 min)
4	98.84	17.55	337.0727	6	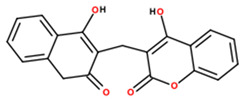	Dicoumarol; (4-hydroxy-3-[(4-hydroxy-2-oxochromen-3-yl)methyl]chromen-2-one)
5	81.14	3.34	317.0717	−95.5	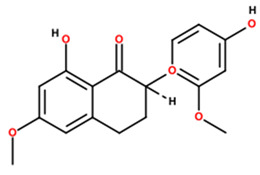	Cajanol; (5-hydroxy-3-(4-hydroxy-2-methoxyphenyl)-7-methoxy-2,3-dihydrochromen-4-one)
6	34	16.61	233.1669	56.9	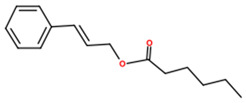	Cinnamyl caproate; ([(*E*)-3-phenylprop-2-enyl] hexanoate)
7	35.49	10.84	161.1179	4.2	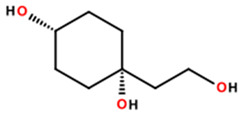	Rengyol; (1-(2-hydroxyethyl)cyclohexane-1,4-diol)
8	100	11.13	323.1379	31.2	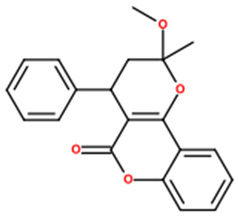	Cyclocumarol; (2-methoxy-2-methyl-4-phenyl-3,4-dihydropyrano [3,2-c]chromen-5-one)
9	36.77	0.48	273.0413	7.2	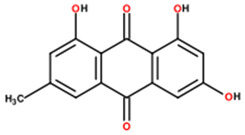	Rheoemodin; (1,3,6,8-tetrahydroxyanthracene-9,10-dione)
10	100	8.37	303.0076	−19.7	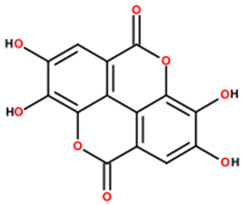	Ellagic acid; (6,7,13,14-tetrahydroxy-2,9-dioxatetracyclo[6.6.2.04,16.011,15]hexadeca-1(15),4,6,8(16),11,13-hexaene-3,10-dione)
11	92.62	0.56	375.1744	81.5	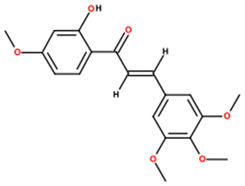	2’-hydroxy-3,4,4’,5,6’-pentamethoxychalcone; (*E*)-1-(2-hydroxy-4,6-dimethoxyphenyl)-3-(3,4,5-trimethoxyphenyl)prop-2-en-1-one)
12	38.92	3.79	389.1223	−2	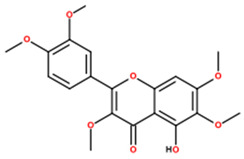	Artemetin; (2-(3,4-dimethoxyphenyl)-5-hydroxy-3,6,7-trimethoxychromen-4-one)

Note: Metabolite assignments correspond to putative level 3–4 annotations obtained via untargeted UPLC-QTOF-MS based on retention time, accurate mass, isotope distribution, and MS/MS fragmentation patterns, using predefined acceptance criteria and supported by searches against LuMet-TCM, Animal_DB, and Herb DB, as well as comparison with the literature. Compound assignments were accepted when the overall identification score was ≥40 and the MS/MS fragmentation match score exceeded 50. No targeted analytical confirmation, authentic standard matching, or individual compound isolation was performed. Therefore, these annotations must be interpreted strictly as putative and not as definitive compound identifications. *m*/*z*, mass-to-charge ratio; ppm, parts per million. Chemical structure images were generated using BIOVIA/Draw, Version 25.1.NET (Dassault Systèmes, Vélizy-Villacoublay, France, 2025).

**Table 2:** Anthropometric and biochemical parameters after bagasse (CAB) or hydroethanolic extract (HECAB) supplementation in HFHC-fed rats.

Parameters	CTRL	HFHC	CAB	HECAB
Energy intake (kJ/day)	387.9 ± 35.8	410.1 ± 48.6 *	388.4 ± 53.4 α	381.0 ± 48.8 α
Food consumption (g/day)	27.9 ± 2.5	21.7 ± 2.5 *	20.6 ± 2.8	20.2 ± 2.5
Final body weight (g)	468.2 ± 35.6	449.7 ± 31.2	373.2 ± 30.9 α	414.7 ± 31.9
Waist circumference (cm)	19.0 ± 1.3	18.21 ± 0.7	16.5 ± 1.1	17.5 ± 0.8
Nose-anus length (cm)	25.6 ± 0.5	25.1 ± 0.5	24.0 ± 0.7	25.0 ± 0.7
Lee Index	0.304 ± 0.0	0.305 ± 0.0	0.301 ± 0.0	0.307 ± 0.0
Triglycerides (mg/dL)	34.3 ± 5.6	35.6 ± 2.5	31.5 ± 1.9	32.0 ± 1.8
Total cholesterol (mg/dL)	67.6 ± 4.2	82.3 ± 6.5 *	87.2 ± 5.3	86.3 ± 2.5
Glucose (mg/dL)	75.6 ± 9.7	108.7 ± 14.1 *	120.6 ± 2.5 α	118.3 ± 3.5
Insulin (UI/mL)	4.8 ± 1.0	8.9 ± 0.5 *	5.8 ± 0.7 α	5.2 ± 1.6 α
HOMA-IR	1.0 ± 0.0	2.5 ± 0.4 *	1.3 ± 0.2 α	1.2 ± 0.4 α

Values are expressed as mean ± SD. Different superscript symbols (* and α) within the same row denote statistically significant differences between groups, according to one-way ANOVA followed by Tukey’s post hoc test (*p* < 0.05). * *p* < 0.05 vs. CTRL; α *p* < 0.05 vs. HFHC. Abbreviations: CTRL, standard diet; HFHC, high-fat, high-carbohydrate diet; CAB, HFHC diet supplemented with cashew apple bagasse; HECAB, HFHC diet supplemented with hydroethanolic extract of cashew apple bagasse; HOMA-IR, homeostatic model assessment of insulin resistance.

## Data Availability

The authors confirm that the data supporting the findings of this study are available within the article. The datasets of this study are available from the corresponding authors upon reasonable request.

## Abbreviations

ABTS, 2,2′-azinobis(3-ethylbenzothiazoline)-6-sulfonic acid; AOAC, Association of Official Analytical Chemists; ARE, antioxidant response element; ARRIVE 2.0, Animal Research: Reporting of In Vivo Experiments guidelines; CAB, cashew apple bagasse; CAT, catalase; CO, carbon monoxide; CPA, collagen proportional area; CTRL, control group; DAB, 3,3′-diaminobenzidine; DPPH, 2,2-diphenyl-1-picrylhydrazyl; ESI, electrospray ionization; GAE, gallic acid equivalents; GAPDH, glyceraldehyde-3-phosphate dehydrogenase; GPX1, glutathione peroxidase 1; H&E, hematoxylin and eosin; HECAB, hydroethanolic extract of cashew apple bagasse; HFHC, high-fat, high-carbohydrate; HO-1, heme oxygenase-1; HOMA-IR, homeostatic model assessment of insulin resistance; HRP, horseradish peroxidase; IL-1β, interleukin-1 beta; IL-6, interleukin-6; IL-10, interleukin-10; MASLD, metabolic dysfunction-associated steatotic liver disease; MASH, metabolic dysfunction-associated steatohepatitis; MSE, data-independent acquisition mode; NF-κB, nuclear factor kappa B; NQO1, NAD(P)H quinone oxidoreductase 1; NRF2, nuclear factor erythroid 2-related factor 2; PGC-1α, peroxisome proliferator-activated receptor gamma coactivator 1-alpha; PVDF, polyvinylidene fluoride; RCF, relative centrifugal force; RIA, radioimmunoassay; ROS, reactive oxygen species; SOD1, superoxide dismutase 1; TE, Trolox equivalents; TNF-α, tumor necrosis factor alpha; TPC, total phenolic content; UPLC-QTOF-MS, ultra-performance liquid chromatography coupled to quadrupole time-of-flight mass spectrometry; UV-Vis, ultraviolet–visible spectrophotometry.
